# Activation of the Anti-Oxidative Stress Response Reactivates Latent HIV-1 Through the Mitochondrial Antiviral Signaling Protein Isoform MiniMAVS

**DOI:** 10.3389/fimmu.2021.682182

**Published:** 2021-06-14

**Authors:** Indra Sarabia, Camille L. Novis, Amanda B. Macedo, Hiroshi Takata, Racheal Nell, Juyeon C. Kakazu, Robert L. Furler, Binita Shakya, Heidi L. Schubert, Christopher P. Hill, Ana Beatriz DePaula-Silva, Adam M. Spivak, Lydie Trautmann, Vicente Planelles, Alberto Bosque

**Affiliations:** ^1^ Department of Microbiology, Immunology and Tropical Medicine, George Washington University, Washington, DC, United States; ^2^ Department of Pathology, Division of Microbiology and Immunology, University of Utah School of Medicine, Salt Lake City, UT, United States; ^3^ Vaccine and Gene Therapy Institute, Oregon Health and Science University, Portland, OR, United States; ^4^ Department of Medicine, Division of Infectious Diseases, University of Utah, Salt Lake City, UT, United States; ^5^ Department of Medicine, Division of Infectious Diseases, Weill Cornell Medicine, New York, NY, United States; ^6^ Department of Biochemistry, University of Utah, Salt Lake City, UT, United States; ^7^ Department of Pharmacology and Toxicology, College of Pharmacy, University of Utah, Salt Lake City, UT, United States

**Keywords:** Dynasore, HIV-1, shock and kill, latency, latency-reversal agents, MAVS, reactive oxygen species, anti-oxidative stress response

## Abstract

The mitochondrial antiviral signaling protein (MAVS) is part of the cell’s innate immune mechanism of defense. MAVS mRNA is bicistronic and can give rise to a full length-MAVS and a shorter isoform termed miniMAVS. In response to viral infections, viral RNA can be sensed by the cytosolic RNA sensors retinoic acid-inducible gene I (RIG-I) and/or melanoma differentiation-associated protein 5 (MDA5) and activate NF-κB through interaction with MAVS. MAVS can also sense cellular stress and activate an anti-oxidative stress (AOS) response through the activation of NF-κB. Because NF-κB is a main cellular transcription factor for HIV-1, we wanted to address what role MAVS plays in HIV-1 reactivation from latency in CD4 T cells. Our results indicate that RIG-I agonists required full length-MAVS whereas the AOS response induced by Dynasore through its catechol group can reactivate latent HIV-1 in a MAVS dependent manner through miniMAVS isoform. Furthermore, we uncover that PKC agonists, a class of latency-reversing agents, induce an AOS response in CD4 T cells and require miniMAVS to fully reactivate latent HIV-1. Our results indicate that the AOS response, through miniMAVS, can induce HIV-1 transcription in response to cellular stress and targeting this pathway adds to the repertoire of approaches to reactivate latent HIV-1 in ‘shock-and-kill’ strategies.

## Introduction

Human Immunodeficiency Virus (HIV) is known to establish a latent infection in antiretroviral therapy (ART) treated individuals (1–3). Elimination or control of this latent reservoir is a critical endpoint to generate either a sterilizing or a functional cure for HIV. “Shock-and-kill” is one of the most prominent cure strategies currently in development to achieve a sterilizing cure for HIV-1. This strategy is based on eliciting viral gene expression from latently infected cells, thus allowing for elimination of latently infected cells by virus-mediated cytopathicity or immune-mediated removal by natural killer, CD8 T cells or other immune effectors. Several latency reversing agents (LRAs) have been developed towards “Shock-and-kill” strategies [for reviews on LRAs see (4–6)].

Pathogen recognition receptors (PRRs) are sensors of the innate immune system and detect pathogen-associated molecular patterns (PAMPs) of bacteria, parasites, fungi or viruses. In addition, they recognize danger-associated molecular patterns (DAMPs), such as nuclear and cytosolic cell components that are released or mislocalized due to tissue damage (7). PRRs can be classified into Toll-like receptors (TLRs), retinoic acid-inducible gene-I (RIG-I)-like receptors (RLRs) and nucleotide-binding oligomerization domain (NOD)-like receptors (NLRs). TLR agonists reactivate latent HIV, increase immune activation and promote antiviral responses [for a review see (8)]. Several TLR agonists, including the TLR3-agonist Poly-ICLC, the TLR7-agonist vesatolimod and the TLR9-agonist MGN1703, have already reached clinical trials for HIV eradication (9–12). Whether RLR agonists can reactivate latent HIV-1 has not been fully explored. A recent work demonstrated that acitretin, a retinoic acid derivative, could reactivate latent HIV-1 and increase levels of RIG-I in a p300-dependent manner. This increase facilitates HIV-1 DNA reduction after reactivation (13). However, these results were unable to be reproduced by another group (14). Regardless, these studies did not address whether activation of RIG-I can directly reactivate latent HIV-1 in CD4 T cells.

In this study, we demonstrate that RIG-I agonists can reactivate HIV-1 and that pharmacological manipulation of MAVS, a downstream adaptor protein of the RLRs RIG-I and MDA-5, can induce HIV-1 reactivation from latency in CD4 T cells. This reactivation is mediated by the generation of reactive-oxygen species (ROS) and the activation of the AOS response through a MAVS dependent mechanism. MAVS mRNA is bicistronic and can produce two functionally distinct proteins (15). The full-length MAVS (FL-MAVS) of 70 kDa, contains a CARD domain and is able to mediate signaling from the PRRs RIG-I and MDA5 to initiate antiviral innate immunity. An internal start codon encodes for a shorter isoform with no CARD domain (miniMAVS). The absence of the CARD domain renders miniMAVS unable to transduce signaling coming from RIG-I or MDA-5 but retains its ability to activate NF-κB (16). In particular, we demonstrate that the short isoform miniMAVS is sufficient to sense the AOS response. Furthermore, we uncover that the latency-reversal activity of two well-studied PKC agonists, Ingenol-3,20-dibenzoate (Ingenol) and Bryostatin-1, is partially dependent on the generation of ROS and the activation of MAVS. Our results suggest that miniMAVS and the AOS response may be a potential target for the development of “shock and kill” strategies against the latent HIV-1 reservoir.

## Materials and Methods

### Reagents

The following reagents were obtained through the NIH AIDS Reagent Program, Division of AIDS, NIAID, NIH: Nelfinavir, human rIL-2 from Dr. Maurice Gately, Hoffmann-La Roche Inc (17). and pNL4-3 from Dr. Malcolm Martin (18). Bryostatin-1 and Ingenol 3,20 dibenzoate were obtained from Enzo Life Sciences. Panobinostat, Romidepsin, SAHA, PFI-1, JQ-1, Bromosporine, Gö6893, Bay 11-7082 and BAPTA were obtained from Cayman Chemical. PMA and Poly(I:C) LMW and Poly(I:C) LMW-Lyovec was obtained from Invivogen. TNF-α was obtained from Peprotech. N-acetyl Cysteine, Catechol, and Prostratin were obtained from Sigma. Dihydroethidium dye was obtained from Invitrogen. CD3/CD28 Dynabeads™ were obtained from ThermoFisher Scientific.

### Cell Lines

2D10 cells were provided by Dr. Jonathan Karn. The 2D10 cells contain a HIV-1 genome with GFP inserted in the place of nef and carry the H13L mutation in Tat that helps to promote proviral entry into latency but still allows HIV-1 transcription (19). J-Lat 10.6 and J-Lat 6.3 cells were provided by Dr. Eric Verdin and contain a full-length HIV-1 genome with a frameshift in *env* that restricts the insert from expressing env or nef (20). J-Lat 5A8 were provided by Dr. Warner Greene and were selected to be responsive to αCD3/αCD28 stimulation (21). All cell lines were cultured in RPMI 1640 with 10% FBS, 1% penicillin-streptomycin and 1% L-glutamine (Invitrogen). Cell cultures were maintained at 37°C under 5% CO_2_.

### Generation of Latently Infected Cultured T_CM_

Naïve CD4^+^ T cells were isolated *via* negative selection from peripheral blood mononuclear cells (PBMC) from healthy donors. Cultured T_CM_ were generated and infected as previously described (22, 23).

### Stimulation of Cells

For J-Lat, 1x10^5^ cells were left untreated or treated with DMSO (Fisher Scientific, Hampton, NH), 25µM Dynasore unless otherwise indicated (Santa Cruz Biotechnology, Santa Cruz, CA), 5ng/mL phorbol-myristate-acetate (PMA), 20µg/ml of Poly(I:C) LMW, 50ng/ml Poly(I:C) LMW-Lyovec (Invitrogen), 10ng/mL TNF- α (Peprotech, Rocky Hill, NJ), 10µM Prostratin (Sigma-Aldrich, Saint Louis, MO), 1µM SAHA, 100nM Bryostatin-1, 100nM Ingenol Dibenzoate (Enzo Life Science, Farmingdale, NY) and 10µM JQ1, 10µM PFI-1, 10µM Bromosporine, 20nM Romidepsin, 15nM Panobinostat, (Cayman Chemicals, Ann Arbor, MI) in triplicates at the concentrations indicated. Three biological replicates were performed per LRA. For the cultured T_CM_ model, 3x10^5^ cells per donor were stimulated as indicated.

For inhibitor studies, cells were pre-incubated with the indicated inhibitors for 2 hours before stimulation.

For inhibitor studies in the cultured T_CM_ model, cells were incubated for 1 hour prior to stimulation with LRAs. 2.5mM NAC or 10µM Gö6893 were used for primary cell experiments. Cells were then stimulated with either 100nM of Bryostatin-1 or Ingenol Dibenzoate, and treated for 48hrs, or CD3/CD28 beads (at a 1:1 ratio of beads to cells) as a positive control.

### Cas9/CRISPR-MAVS Design and Vector Construction

Cas9 target sites were identified using the online CRISPR design tool (crispr.mit.edu) (24, 25). The CRISPR-HSAS plasmid was constructed by cloning HSAS in place of eGFP from an original lentiCRISPRv2 plasmid provided from Dr. Ryan O’Connell’s lab (University of Utah). The Exon 5 sequence of the human MAVS gene was used to generate sgRNAs as follows: 5’-ACAGGGTCAGTTGTATCTAC-3’ and 5’-GTAGATACAACAACTGACCCTGT-3’. These paired oligos included a 4-bp overhang to facilitate cloning into the Bsmb-I site of the lentiviral construct. The resulting plasmid was transformed into One Shot Stbl3 (Life Technologies) and verified by sequencing. A CRISPR-HSAS-Scramble plasmid was also generated using the following pair of oligos, 5’- GGCACTACCAGAGCTAACTCA-3’ and 5’-TGAGTTAGCTCTGGTAGTGCC-3’.

### Generation of J-Lat ΔMAVS Clone

Briefly, to make lentiviruses, CRISPR-MAVS-HSA was co-transfected with the pVSVg pseudotyping plasmid and the psPAX2 packaging plasmid into HEK293T cells. After 48 hours, supernatant-containing viruses was pelleted by ultracentrifugation. J-Lat clone 10.6 was infected with CRISPR-HSAS viruses by spinoculation. 10 days after infection, HSA+ cells were isolated by flow cytometry sorting (FACS ARIA) and expanded. A single cell clone was achieved by plating 0.5 cell suspended in RPMI media per well in 96 well plates. Clones were detected by microscopy after 7 days and transferred to 12 well plates. MAVS knockout was assessed by Western blot.

### MAVS Complementation

A bicistronic lentiviral vector derived from pFIN-EF1-GFP-2A-mCherry-WPRE (kindly provided by Dr. Susan L. Semple-Rowland at the University of Florida McKnight Brain Institute) was engineered to express MAVS in place of GFP (26). MAVS was synthetically generated using Integrated DNA Technologies. pFIN-MAVS containing vector and empty vector were transfected into 293FT cells to generate lentivirus. 500,000 cells of the J-Lat ΔMAVS clone were transduced with empty vector (no MAVS) or pFIN-MAVS by spinoculation at 37C, 2900 RPM for 2 hours. Virus was removed and cells transduced were plated at 125,000 per ml in complete media for 48 hours. Cells transduced with pFIN-MAVS were also incubated in the presence of caspase inhibitor ZVAD-FMK (Apex Bio), as overexpression of MAVS has been shown to induce apoptosis (27). After 48 hours, cells were washed to remove ZVAD-FMK and were plated at 50,000 per well in triplicates and incubated with LRAs, at the concentrations previously indicated, for 24 hours. Cells were fixed, stained, and assessed for reactivation and viability by flow cytometry. Transduction with empty vector virus or pFIN-MAVS virus was measured by mCherry expression by flow cytometry.

### Flow Cytometry Analysis

To assess intracellular p24Gag expression, 1×10^5^ cells were fixed, permeabilized, and stained as previously described (22). In all experiments, HIV-1 p24Gag negative staining regions were set with uninfected cells treated in parallel.

GFP and mCherry fluorescence were measured in a BD FACSCanto II analyzer and cells were sorted in a BD FACSAria II. Expression was measured in a BD FACSCanto II flow cytometer. Data was analyzed using FlowJo (TreeStar Inc, Ashland, OR).

To analyze mitochondrial profile, thawed PBMCs from a healthy individual donor and WT and ΔMAVS J-Lat clones were first rested at 37°C for 10 minutes, and were stained with MitoTracker Green, MitoTracker Orange CM-H_2_TMRos, and CellROX Deep Red according to the manufacture’s protocol (Thermo Fisher Scientific, Molecular Probes). After two times washing with RPMI-1640 supplement with 10% FBS, the cells were stained with cell surface markers, Annexin V, and LIVE/DEAD Fixable Aqua Dead Cell Stain Kit. The live cells (LIVE/DEAD^-^Annexin V^-^) were analyzed without fixation on FACS Aria II (BD Biosciences).

To analyze phosphorylated p65 and degradation of IκB-α, memory CD4+ T cells were serum-starved overnight in RPMI + 2% FBS. Next day, cells were stimulated for the indicated times at 37°C with 25 μM Cathecol, 100nM Bryostatin-1, 100nM Ingenol Dibenzoate or 200 nM PMA and 1 μM Ionomycin as positive control. After incubation, cells were washed and stained with a viability dye (Fixable Viability Dye eFluor 450, Affymetrix, eBioscience, 65-0863-18) for 10 minutes at 4°C. Cells were then fixed with 100 μl of prewarmed (37°C) Fix Buffer I (Becton Dickinson) for 10 minutes at 37°C. Cells were washed once with 1 ml of PBS containing 3% FBS (PBS/3% FBS). Cells were then permeabilized while vortexing with 100 μl of Perm Buffer III (Becton Dickinson) and incubated for 30 minutes on ice. Cells were washed once with 1 ml of PBS/3% FBS and stained with mouse anti–phosphoserine 529 p65 conjugated to Alexa Fluor 488 (clone K10-895.12.50, catalog 558423, BD Bioscience) and mouse anti- IκB-α conjugated to PE (Clone 25/IkBa/MAD-3, catalog 560818, BD Bioscience) in 100 μl of PBS/3% FBS for 16 hours at 4°C for 1 hour. Finally, cells were washed once with 1 ml of PBS/3% FBS. Cells were analyzed on a BD LSR Fortessa X20 flow cytometer with FACSDIVA software (Becton Dickinson) and analyzed using FlowJo (TreeStar Inc, Ashland, OR).

### Western Blotting

To analyze gene knockdown or knockout, cells were lysed with NETN extract buffer containing 100mM NaCl, 20mM Tris-Cl (pH 8), 0.5mM EDTA, 0.5% Nonidet P-40, protease inhibitor cocktail (cOmplete, Roche) and phosphatase inhibitor cocktail (phosSTOP, Roche) for 30 min on ice. Lysates were cleared by centrifugation at 12,000 rpm for 10 minutes at 4°C. Proteins were visualized on SDS-PAGE. Western blotting was performed according to the standard protocols. The following antibodies were used at the following concentrations: anti-MAVS at a 1:1,000 dilution (Santa Cruz Biotechnology, Santa Cruz, CA), HA at a 1:1000 dilution (Biolegend), anti-PKCα, anti-PKC-δ, anti-PKCµ, anti-PKCζ were each used at a 1:1,000 dilution (Cell Signaling, Danvers, MA), and anti-β-actin antibody at a 1:10,000 dilution (Sigma-Aldrich, Saint Louis, MO). Secondary anti-rabbit and secondary anti-mouse antibodies were used at a 1:10,00 dilution (Jackson ImmunoResearch, #111-035-046 and 115-035-146, respectively). To analyze cytoplasmic and nuclear fractions, samples were prepared as previously described (28) and according to manufacturer instructions using the Qproteome cell compartment kit (Qiagen).

### TransAM NF-κB p65

To analyze activation of p65, cells were lysed with NETN extract buffer. 4 μg of total protein was analyzed using the TransAM™ NFκB p65 Transcription Factor Assay Kit (Active Motif, Inc) following the manufacture protocol.

### Superoxide Assay

Generation of ROS was detected using dihydroethidium dye and flow cytometry. MAVS clones or memory CD4 T cells were washed twice with PBS and 100,000 cells were plated per well in 50uL of RPMI (without serum or supplementation). Cells were treated with LRAs or 100µM TBHP (positive control) for the indicated time and then stained with 2µM Dihydroethidium dye at 37 °C in the dark for 15 minutes. Cells were assayed by flow cytometry immediately after addition of 100uL PBS.

### Seahorse XF Cell Mito Stress Test

WT and ΔMAVS J-Lat clones were seeded at 200,000 cells/well in CELL-TAK (Corning) coated XF96 Cell Culture Microplate. The Seahorse XF Cell Mito Stress Test was performed on XFe96 Analyzer according to manufacturer’s protocol (Agilent). OCR values for Basal, Proton Leak, ATP Production, Non-mitochondrial Respiration, Maximal Respiration, and Spare Respiratory Capacity were calculated with Seahorse XF Stress Test Report Generator within Wave software (Agilent).

### Participant Involvement

Cells from uninfected blood donors. Blood donors 18 years and older serve as blood donors. Written informed consent was obtained from all donors. These studies are covered under the institutional review board (IRB) #67637 protocol approved by the University of Utah Institutional Review Board or blood was obtained from the Gulf Coast Regional Blood Center (Houston, TX). Cells from infected HIV-1^+^ donors (REVEAL assay). Aviremic people living with HIV-1(PLWH) on ART were recruited for phlebotomy according to an approved IRB protocol #58246 at the University of Utah. Inclusion criteria for this study required viral suppression (<50 HIV-1 RNA copies/ml) for a minimum of 6 months, ART initiation during chronic HIV-1 infection (>6 months since seroconversion), and compliance with a stable ART regimen for a minimum of 12 months per participant and provider reports. Informed consents were obtained, and phlebotomies were performed in the Center for Clinical and Translational Science Clinical Services Core at the University of Utah Medical Center. All research was performed in accordance with relevant guidelines/regulations.

### Statistics

Two-tailed paired-samples t-test analysis or Wilcoxon matched-pairs signed rank test was used to calculate p-values. When appropriate, column statistics and p-values were calculated. p-values were calculated using Prism 5 for Mac OS X software (GraphPad Software, Inc., La Jolla, CA).

## Results

### Dynasore Reactivates Latent HIV-1 in Different *In Vitro* Models of Latency and in Cells Isolated From Aviremic People Living With HIV (PLWH)

Dynasore or 3-hydroxy-naphthalene-2-carboxylic acid (3,4-dihydroxy-benzylidene)-hydrazide is a potent inhibitor of dynamin-dependent endocytosis (29). Besides its ability to inhibit dynamin-related processes, Dynasore has other activities that are independent of dynamin (30, 31). In particular, Dynasore induces activation of the transcription factor NF-κB by activating the mitochondrial antiviral signaling protein (MAVS) (32). This process is mediated by the generation of reactive oxygen species (ROS), and it is independent of Dynasore’s ability to inhibit endocytosis. It is well established that NF-κB induces HIV-1 transcription (33). Therefore, we hypothesized that pharmacological activation of MAVS would induce the reactivation of latent HIV-1 through an NF-κB dependent mechanism, and that targeting MAVS may represent a novel strategy toward HIV-1 cure efforts involving “shock and kill” strategies.

**Figure 1:**
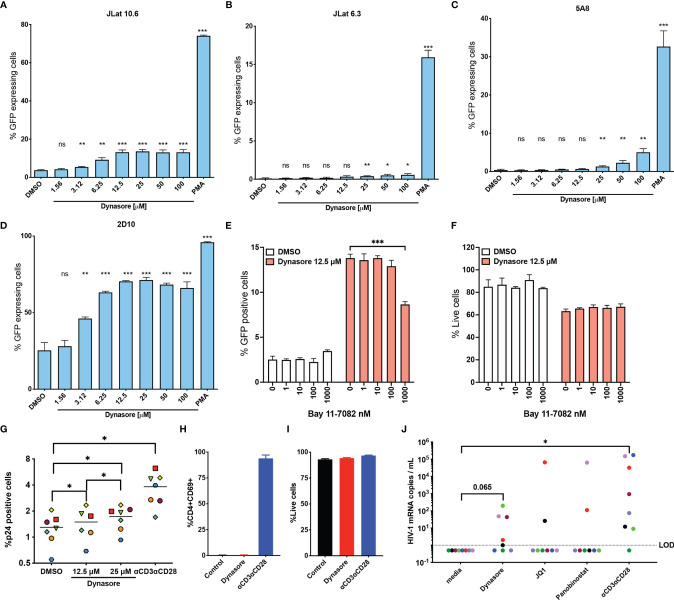
Dynasore reactivates latent HIV-1 in *in vitro* models of HIV-1 latency and cells isolated from aviremic HIV participants. **(A)** J-Lat 10.6, **(B)** J-Lat 6.3, **(C)** 5A8 and **(D)** 2D10 cell lines were treated with increasing concentrations of Dynasore or with 5 ng/ml PMA. Percentage of cells expressing GFP was assessed by flow cytometry. Bar graph corresponds to mean and standard deviation of experiments performed in triplicates. Significance was calculated by unpaired t test analysis. J-Lat10.6 was tested in their ability to reactivate latent HIV-1 with Dynasore in the presence of the NF- κB inhibitor Bay 11-7082. % GFP positive cells **(E)** and viability **(F)** were measured by flow cytometry. Data are represented as mean of triplicate replicates. Unpaired t-test relative to no inhibitor was used to calculate p-values. **(G)** Latently infected cultured T_CM_ were treated with solvent DMSO, 12.5 μM and 25 μM Dynasore or αCD3/αCD28 beads. Percentage of cells expressing p24-gag was assessed by flow cytometry. Experiments were done with cells generated from 7 different donors. Each symbol corresponds to a different donor. Mean is indicated with a horizontal line. Statistically significant p values determined by using Wilcoxon matched pairs signed rank test analysis relative to media control. Memory CD4 T cells from three HIV-negative donors were treated with 12.5 μM Dynasore and levels of CD4 activation **(H)** and toxicity **(I)** were measured at 48h by flow cytometry. **(J)** Viral reactivation mediated by 12.5 μM Dynasore, 200 nM JQ-1, 10 nM Panobinostat or αCD3/αCD28 was measured in cells isolated from 8 aviremic PLWH using the REVEAL assay. Wilcoxon matched-pairs signed rank test analysis relative to media control was used to calculate p-values. (*< 0.05, **< 0.01, ***< 0.001, ns, not significant).

To test our hypothesis, we first evaluated whether Dynasore could reactivate latent HIV-1 in four different models of latency derived from the transformed cell line Jurkat (**Figures 1A–D**). We first assessed the activity of this compound in two J-Lat clones (6.3 and 10.6) (20). In J-Lat 10.6, Dynasore reactivated HIV-1 in a dose dependent manner, reaching a plateau at 12.5 µM (**Figure 1A**). In J-Lat 6.3, Dynasore induced modest HIV-1 reactivation at concentrations ranging from 25 μM to 100 μM (**Figure 1B**). We also tested whether Dynasore could reactivate latent HIV-1 in the J-Lat clone 5A8 (21) (**Figure 1C**). This clone was obtained *via* selection for increased responsiveness to αCD3/αCD28 antibodies (21, 34). In this cell line, Dynasore reactivated latent HIV-1 at concentrations higher than 25 μM. Finally, we tested the cell line 2D10 (19). This cell line carries a provirus with a hypomorphic mutation in Tat (H13L), that promotes viral entry into latency while still allowing HIV-1 transcription to be inducible. As shown in **Figure 1D**, Dynasore induced viral reactivation in latently infected 2D10 cells in a dose-dependent manner, reaching a plateau at 12.5 µM. Dynasore has been shown to activate NF-κB through the induction of ROS and subsequent activation of MAVS (32). We confirmed that viral reactivation mediated by Dynasore was dependent on NF-κB. The addition of the NF-κB specific inhibitor Bay 11-7082 reduced viral reactivation in a dose-dependent manner in the absence of overt toxicity (**Figures 1E, F**).

We then assessed whether Dynasore reactivated latent HIV-1 in primary cells using the cultured T_CM_ model of latency (22, 23, 35). As shown in **Figure 1G**, Dynasore was able to reactivate latent HIV-1 in this model generated from 7 donors in a dose dependent manner over the DMSO control. In this primary cell model of latency, Dynasore reactivated an average of 7.8% at 12.5 μM and 16.9% at 25 μM relative to that of αCD3/αCD28 relative to DMSO control. We then tested whether Dynasore could lead to T cell activation. In contrast with αCD3/αCD28, Dynasore did not induce CD4 T cell activation as measured by the induction of the early activation marker CD69 (**Figure 1H**) nor was it toxic in primary CD4T cells at the time and concentration tested (**Figure 1I**). This is in contrast with what was observed in the transformed cell line, in which Dynasore presented some toxicity (**Figure 1F**). Finally, we tested the ability of Dynasore to reactivate latent HIV-1 in cells isolated from aviremic PLWH using the Rapid *Ex-Vivo* Evaluation of Anti-Latency assay (REVEAL) (36). This assay was designed to amplify genomic RNA from virions in culture supernatant. Resting CD4 T cells were isolated from aviremic PLWH and were cultured for 48 hours in the presence or absence of LRAs. Afterwards, culture supernatant was collected to measure HIV-1 mRNA using qPCR (36). In this assay, we detected viral release over untreated control in 5 out of 8 PLWH when treated with Dynasore while we could only detect 2 out of 8 with the LRAs JQ-1 or Panobinostat. αCD3/αCD28 induced reactivation in 7 out of 8 PLWH (**Figure 1J**).

**Figure 2:**
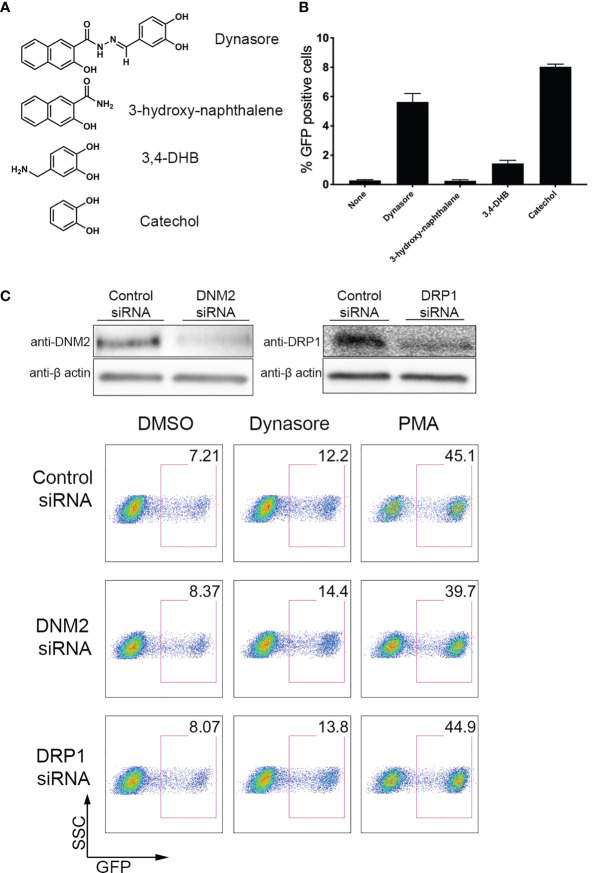
Dynasore Structure Activity Relationship and dynamin-independent reactivation of latent HIV-1. **(A)** Chemical structures of components of Dynasore. **(B)** J-Lat 10.6 cells treated with 10µM Dynasore, Hydroxynaphthamide, 3,4-dihydroxybenzylamide (3,4-DHB) or Catechol for 24 hours. % GFP positive cells were measured by flow cytometry. Experiments were performed in triplicate. Data are represented as mean ± SD. **(C)** Immunoblot analysis of dynamin-2 (DNM2) and drp1 knockdowns in J-LAT 10.6 cell line. Control scramble siRNA and siRNA against dynamin-2 and drp-1 were introduced into J-LAT cell by Amaxa Nucleofection twice. After 24h, cells were either lysed for western blot analysis or incubated with/out solvent DMSO, 25μM Dynasore or 5 ng/ml PMA for 24h and assessed for viral reactivation by flow cytometry. Data are representative of two independent experiments.

We next wanted to address what is the chemical entity of Dynasore responsible for reactivating HIV-1. Dynasore can be separated into 3-hydroxy-naphthalene and 3,4-dihydroxybenzylamine (3, 4-DHB) (**Figure 2A**). We tested these two compounds in J-Lat 10.6 and found that only 3,4-DHB had some activity toward reactivating latent HIV-1 (**Figure 2B**). 3,4-DHB main chemical group is catechol (pyrocatechol or 1,2-dihydroxybenzene), so we next tested whether the catechol group is sufficient to reactivate latent HIV-1. As it is shown in **Figure 2B**, the catechol group is sufficient to reactivate latent HIV-1. Dynasore was previously reported to induce MAVS and NF-κB activation independently of dynamin. We then knocked down expression of dynamins DNM2 and DRP1, and then stimulated J-Lat with Dynasore or our positive control, PMA (a phorbol ester, potent activator of NF-κB). We found that DNM2 or DRP1 knockdown did not affect Dynasore- or PMA-mediated latent HIV reactivation (**Figure 2C**). Taken together, these results indicate that Dynasore, through its catechol group and independently of dynamin inhibition, has the ability to reactivate latent HIV-1 in the J-Lat model of latency and in primary CD4T cells.

### MAVS Is Necessary for Viral Reactivation Mediated by Dynasore and Its Catechol Group

**Figure 3:**
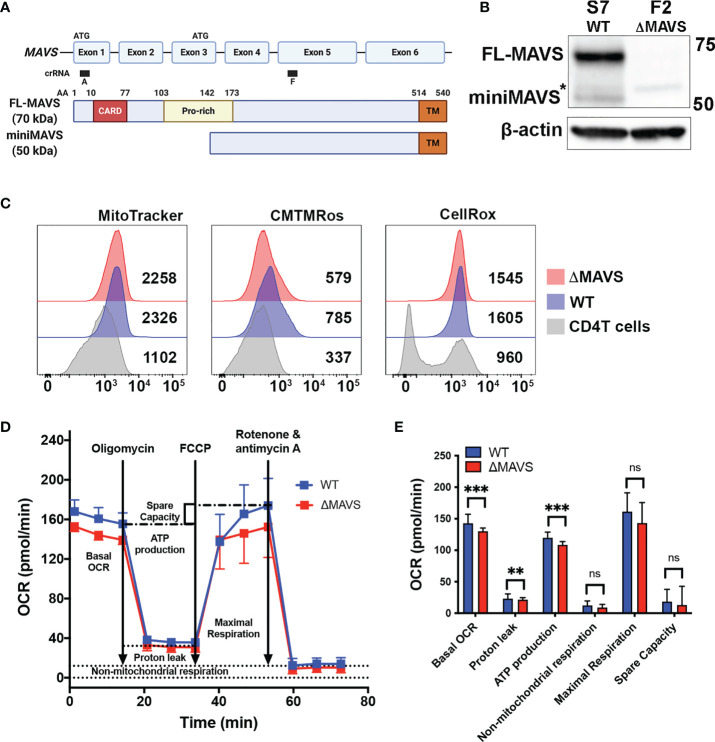
Characterization of J-Lat ΔMAVS. **(A)** Exon and protein map of MAVS and CRISPR guide RNA targeting regions. Created with BioRender.com **(B)** Western blot of selected CRISPR clones generated with a scrambled guide RNA (S7-WT) or generated targeting exon 5 of MAVS (F2-ΔMAVS). *non-specific band. **(C)** Analysis of mitochondrial content (MitoTracker), mitochondrial membrane potential (CMTMRos) and reactive oxygen species content (CellROX) in the WT and ΔMAVS J-Lat clone and compare with that of primary CD4 T cells. **(D, E)** Analysis of mitochondrial function or Oxygen Consumption Rate (OCR) in WT and ΔMAVS J-Lat clone using the Agilent Seahorse XF Cell Mito Stress Test. Data are represented as mean ± SD of 21 measurements. Mann-Whitney test was used to calculate p-values between WT and ΔMAVS J-Lat clone. (**p < 0.01; ***p < 0.001; ns, not significant).

To explore the role of MAVS in viral reactivation mediated by Dynasore, we used a CRISPR-Cas9 strategy to knock-out MAVS expression in J-Lat 10.6. We designed a lentiviral vector with a single guide RNA (gRNA) targeting exon 5 (denoted as “F” clones) of MAVS (**Figure 3A**). After infection of J-Lat 10.6, cells were sorted, and single cell clones were generated. The lack of MAVS was then confirmed by Western blot. We selected two clones for our experiments. S7 is a clone transduced with a scrambled gRNA (Wild Type, WT). In the S7 clone, MAVS expression was not affected (**Figure 3B**) but it has gone through the same single-cell selection process. Clone F2 was generated from cells transduced with the gRNA directed to exon 5 of MAVS. This clone lacks the expression of both FL-MAVS and miniMAVS (from now on referred as ΔMAVS) (**Figure 3B**).

Because MAVS is primarily a mitochondrial protein, we investigated whether the absence of MAVS alters mitochondrial stability and function. First, we addressed whether the absence of MAVS affects mitochondrial content. Both WT and ΔMAVS clones were stained with the green-fluorescent mitochondrial stain MitoTracker Green FM in parallel with human primary CD4^+^ T cells. MitoTracker Green FM localizes to mitochondria regardless of its membrane potential. Both clones showed similar mitochondrial content (**Figure 3C**, MitoTracker) albeit to a higher degree than primary CD4^+^ T cells. This is not unexpected as Jurkat is a transformed cell line whose energetic demand is much higher than that of primary CD4^+^ T cells. Second, we analyzed mitochondrial membrane potential using MitoTracker Orange CM-H_2_TMRos. Lack of MAVS slightly reduced the mitochondrial membrane potential relative to the WT clone (**Figure 3C**, CMTMRos). As for the mitochondrial content, both clones have a higher membrane potential than that of primary CD4^+^ T cells. Third, we measured reactive oxygen species (ROS) content using the CellROX Deep Red. Both clones had similar ROS content but drastically higher than primary CD4^+^ T cells (**Figure 3C**, CellRox). Interestingly, two distinct populations could be observed in primary CD4T cells, probably due to different activation stages. Finally, we addressed whether the absence of MAVS had an effect on mitochondrial function by measuring the Oxygen Consumption Rate (OCR) with the Agilent Seahorse XF Cell Mito Stress Test (**Figure 3D**). This test first measured the basal respiration rate of each clone which indicates the oxygen consumption used to meet cellular ATP demand. The ΔMAVS clone showed a lower basal respiration rate than WT at baseline (**Figures 3D, E**). This result suggests a lower energetic demand for the ΔMAVS clone. To address how much of the OCR is due to ATP synthesis by the mitochondria, the ATP synthase inhibitor oligomycin was injected into the system. The absence of MAVS in the mitochondria slightly reduced ATP production driven respiration (**Figures 3D, E**), suggesting a minor role of MAVS in ATP production. Next, the mitochondrial uncoupler FCCP was added into the system. FCCP uncouples mitochondrial membrane potential and allows for the measurement of maximal respiration to assess how the cells react to increased ATP demand. No differences were observed between both clones (**Figures 3D, E**). Finally, a combination of rotenone and antimycin A was added to completely inhibit mitochondrial respiration, to determine the non-mitochondrial sources of oxygen consumption. The absence of MAVS did not modify the non-mitochondrial respiration (**Figures 3D, E**). The non-mitochondrial respiration is also used to calculate the proton leak of the mitochondria that is responsible for the generation of heat and not ATP. The absence of MAVS slightly reduced proton leak from the mitochondria (**Figures 3D, E**). Finally, the difference between the maximal respiration and the basal respiration is used to calculate the spare capacity. No differences between both cell clones were observed (**Figures 3D, E**). In conclusion, the absence of MAVS is not lethal for Jurkat cells but had minor effects on mitochondrial potential as well as ATP production, suggesting that MAVS may have other roles in energy biogenesis and mitochondrial function besides being an adaptor protein for RIG-I and MDA-5 and the antiviral response.

**Figure 4:**
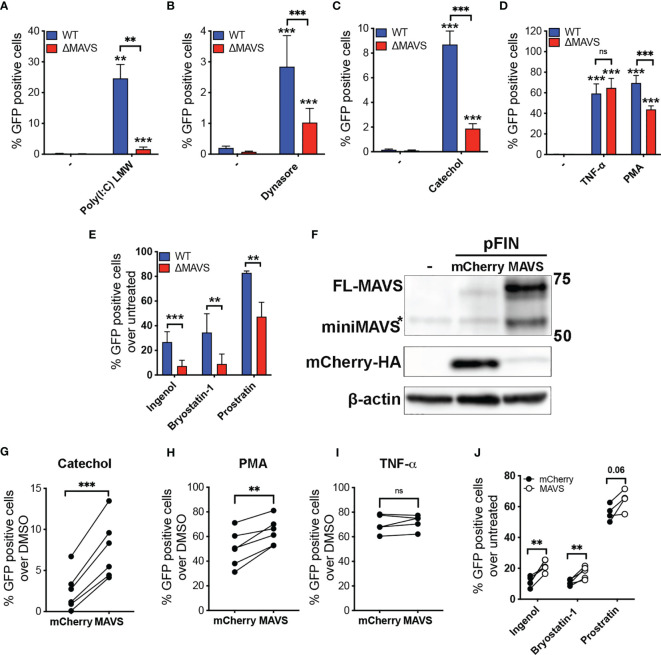
MAVS is necessary for efficient viral reactivation mediated by RIG-I agonists, Dynasore, Catechol and PKC agonists. J-Lat-WT and J-Lat**-**ΔMAVS S7 and F2 clones were tested in their ability to reactivate latent HIV-1 with transfected RIG-I agonist Poly(I:C) LMW **(A)**, Dynasore **(B)**, Catechol **(C)**, TNF-α or PMA **(D)** or Ingenol, Bryostatin-1 and Prostratin **(E)**. % GFP positive cells were measured by flow cytometry. Data are represented as mean ± SD of five biological replicates performed in triplicates. Mann-Whitney test was used to calculate p-values between WT and ΔMAVS J-Lat clone. **(F)** J-Lat**-**ΔMAVS was complemented using a lentiviral vector containing MAVS or an empty vector. Levels of MAVS were analyzed by western. MAVS-transduced or control mCherry-transduced J-Lat**-**ΔMAVS cells were treated for 24 hours with either Catechol **(G)**, PMA **(H)**, TNF-α **(I)**, or Ingenol, Bryostatin-1 and Prostratin **(J)**. % GFP positive cells were measured by flow cytometry. Data are represented as mean of five or six biological replicates perform in triplicates. Two-sample paired t-test analysis was used to calculate p-values between MAVS-transduced or control mCherry-transduced J-Lat**-**ΔMAVS cells. (**p < 0.01; ***p < 0.001; ns, not significant).

Next, WT and ΔMAVS clones were tested for their ability to reactivate latent HIV-1. We first tested a RIG-I agonist. We transfected WT and ΔMAVS clones with Poly(I:C) (LMW), which is a synthetic dsRNA polymer previously shown to be sensed by RIG-I and MDA-5 and activate NF-κB through MAVS (37). As expected, Poly(I:C) (LMW) was able to reactivate latent HIV-1 only in the WT clone but not in the ΔMAVS clone (**Figure 4A**). We observed that the ΔMAVS clone reactivated latent HIV-1 less effectively with Dynasore or its catechol group than the MAVS WT clone (**Figures 4B, C**). Both Dynasore and the catechol group reactivated latent HIV in a dose dependent manner with similar EC_50_ (**Supplementary Figure 1**). Absence of MAVS drastically reduced the EC_50_ of both compounds.

To confirm that this defect in viral reactivation was specific of MAVS-mediated activation of NF-κB, both clones were treated with Tumor Necrosis Factor α (TNFα) (**Figure 4D**), which activates NF-κB in a MAVS-independent manner. These two clones reactivated latent HIV-1 to a similar degree with TNFα. Unexpectedly, the absence of MAVS had a significant effect in viral reactivation mediated by the PKC agonist and NF-κB activator phorbol 12-myristate 13-acetate (PMA) (**Figure 4D**). The results observed with PMA could be due to differences in PKC expression between the two clones. To address this possibility, we confirmed PKC isoform expression levels by western blot (**Supplementary Figure 2**). There was no difference in expression of PKC isoforms analyzed when comparing the WT clone to ΔMAVS. To confirm whether MAVS-dependent activation of latent HIV occurs with other PKC agonists, we tested Bryostatin-1, Ingenol-3,20-dibenzoate (referred to here as Ingenol), and Prostratin. All these agonists have been shown to reactivate latent HIV-1 in several models of latency as well as in cells isolated from aviremic PLWH (34, 36, 38, 39). As we observed with the PKC agonist PMA, viral reactivation mediated by the PKC agonists Ingenol, Bryostatin-1, and Prostratin was decreased in the absence of MAVS (**Figure 4E**).

The decreased viral reactivation mediated by Dynasore, its catechol group or PKC agonists could be a potential consequence related to off-target effects of the CRISPR/Cas9 strategy or the single cell cloning. To address this possibility, we confirmed that MAVS was required for viral reactivation by complementing MAVS in the ΔMAVS clone. To achieve this, the ΔMAVS clone was transduced with a bicistronic lentiviral vector encoding MAVS and HA-mCherry containing a viral 2A cleavage sequence or mCherry alone (26). After transduction, cells were challenged with either the catechol group, TNF-α or PMA. As shown in **Figure 4F**, lentiviral transduction of a construct encoding MAVS efficiently increased the level of MAVS in the ΔMAVS clone while transduction with mCherry did not. As expected, expression of MAVS rescued viral reactivation mediated by the catechol group (**Figure 4G**) and PMA (**Figure 4H**) but not TNF-α (**Figure 4I**). Complementation of MAVS expression significantly restored the defect in viral reactivation observed with Ingenol and Bryostatin-1 (**Figure 4J**). A similar trend was observed with Prostratin, although statistical significance was not achieved for this agonist (**Figure 4J**). These results confirm the essential role of MAVS in viral reactivation mediated by the catechol group of Dynasore and uncovers the unexpected role of MAVS in viral reactivation mediated by the PKC agonists PMA, Ingenol and Bryostatin-1.

### The Catechol Group of Dynasore Is Sufficient to Activate the Transcriptional Activity of NF-κB

**Figure 5:**
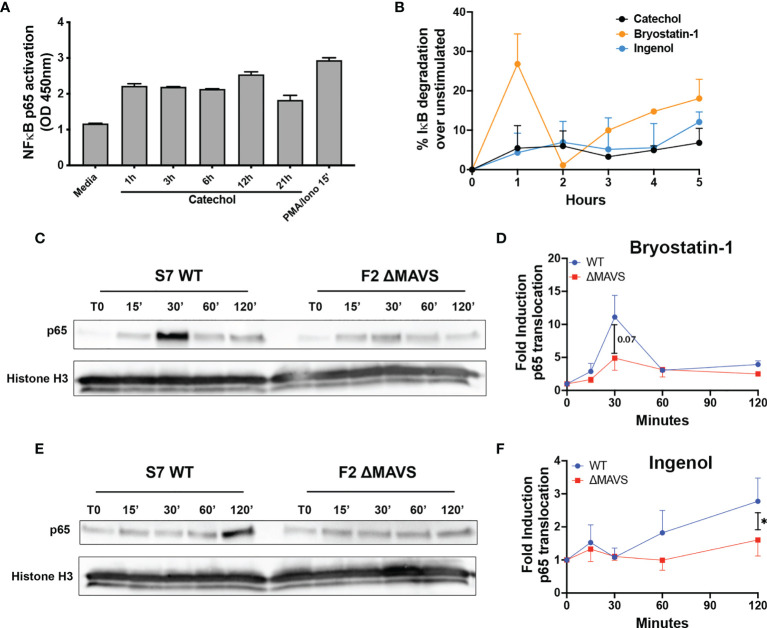
Activation of p65 by Catechol, Bryostatin-1 and Ingenol. **(A)** Total CD4T cells from one donor were treated with 25 μM of Catechol or PMA/Ionomycin at the indicated times. p65 transcriptional activity was measured in total cell extracts using the TransAM NF-κB p65 kit. **(B)** Memory CD4T cells from two donors were treated with Catechol, Ingenol or Bryostatin-1 at the indicated time points and degradation of IκB-α was assessed by flow cytometry. **(C)** Representative western blot of the levels of p65 nuclear translocation after treatment of WT and ΔMAVS J-Lat clone with Bryostatin-1 for the time points indicated. **(D)** Fold induction relative to time 0 (T0) for three independent experiments were quantified by densitometry. **(E)** Representative western blot of the levels of p65 nuclear translocation after treatment of WT and ΔMAVS J-Lat clone with Ingenol for the time points indicated. **(F)** Fold induction relative to time 0 (T0) for three independent experiments were quantified by densitometry. Ratio paired t-test analysis was used to calculate p-values. (*p < 0.05).

Dynasore has been shown to activate NF-κB through MAVS in a RIG-I/MDA-5 independent manner in the murine macrophage cell line RAW 264.7 cells (32). We have shown that the catechol group of Dynasore was sufficient to reactivate latent HIV. A such, we hypothesize that the catechol group will be sufficient to activate NF-κB. To confirm whether the cathecol group could also promote NF-κB activation in primary CD4T cells, we evaluated the activation of the transcriptional activity of NF-κB using the TransAM DNA-binding NF-κB p65 ELISA kit. The ability of the active subunit of NF-κB p65 to bind to an oligonucleotide containing the consensus sequence 5’-GGGACTTTCC-3’ is measured using a colorimetric kit. As shown in **Figure 5A**, catechol was sufficient to increase binding of p65 to the NF-κB consensus sequence albeit to a lower extend than PMA plus Ionomycin.

To further investigate how the catechol group induces NF-κB activation, we measured the ability of the catechol group to promote the degradation of nuclear factor of kappa light polypeptide gene enhancer in B-cells inhibitor, alpha (IκBα) and/or increased the levels of p65 phosphorylation in serine 529 (Ser529) and compared with Ingenol and Bryostatin-1. IκBα binds NF-κB in the cytoplasm and conceals the nuclear localization signal (40). Its degradation is required for efficient p65 nuclear translocation. On the other hand, Ser529 increases p65 transcriptional activity (41, 42). The catechol group promoted IκBα degradation with a slow kinetics similar to Ingenol (**Figure 5B** and **Supplementary Figure 3**). On the other hand, Bryostatin-1 promoted IκBα degradation in a biphasic way. We observed a first strong IκBα degradation, followed by a rescue on expression and a second wave of degradation with slower kinetics (**Figure 5B** and **Supplementary Figure 3**). This is not unexpected as IκBα is a transcriptional target of NF-κB and gets induced upon NF-κB activation (43). Interestingly, although we observed strong Ser529 phosphorylation upon stimulation of CD4T cells with PMA plus Ionomycin, we did not observe Ser529 phosphorylation for either the PKC agonists or the catechol group (**Supplementary Figure 3**). These results suggest that the catechol group can promote the activation of NF-κB by promoting the degradation of IκBα.

### MAVS Is Required for Effective PKC-Agonist Mediated NF-κB Nuclear Translocation

Ingenol and Bryostatin-1 reactivate latent HIV-1 by targeting different PKC isoforms leading to downstream activation of NF-κB, a master transcriptional regulator of HIV-1. Since the absence of MAVS results in a reduction of their LRA activity, we addressed whether the lack of MAVS would lead to a reduction in NF-κB nuclear translocation after stimulation with these two PKC agonists. After stimulation with either Ingenol or Bryostatin-1 at several time points, nuclear translocation of the active subunit of NF-κB p65 was analyzed by western blot. Ingenol and Bryostatin-1 induced nuclear translocation of p65 in the WT clone albeit with different kinetics. Nuclear translocation of p65 was lower in the ΔMAVS clone than the WT clone for both Bryostatin-1 (**Figures 5C, D**) and Ingenol (**Figures 5E, F**). These results further confirm that MAVS is required for efficient NF-κB activation mediated by the PKC agonists Ingenol and Bryostatin-1.

### ROS Are Required for Effective MAVS-Dependent Viral Reactivation Mediated by the Catechol Group, Ingenol and Bryostatin-1

**Figure 6:**
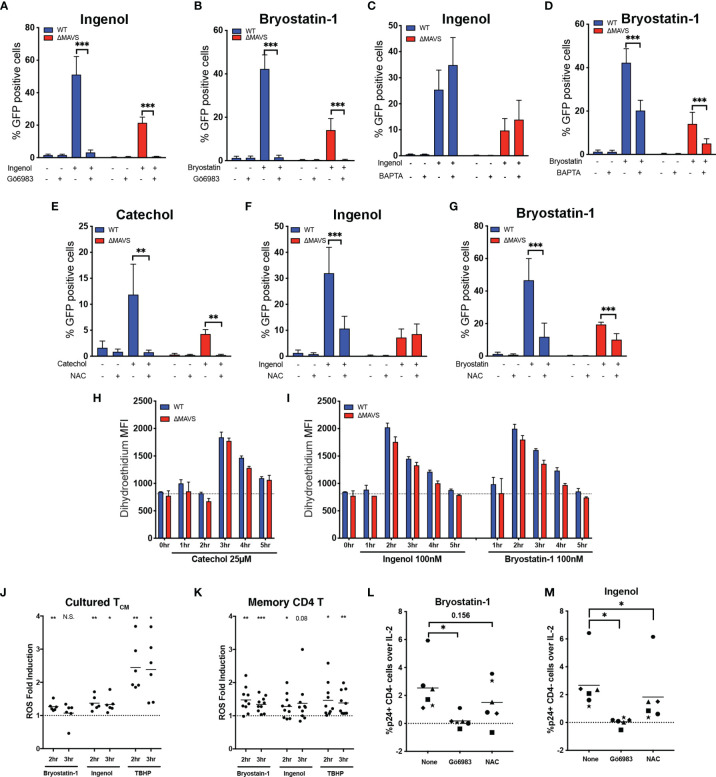
ROS plays a role in viral reactivation mediated by Cathecol and PKC agonists. J-Lat-WT and J-Lat-ΔMAVS clones were tested in their ability to reactivate latent HIV-1 with Ingenol or Bryostatin- in the presence of the PKC inhibitor Gö6983 **(A, B)** or the calcium chelator BAPTA **(C, D)**. J-Lat-WT and J-Lat-ΔMAVS clones were tested in their ability to reactivate latent HIV-1 with Catechol **(E)**, Ingenol **(F)** or Bryostatin-1 **(G)** in the presence of an antioxidant, NAC. % GFP positive cells were measured by flow cytometry. Data are represented as mean ± SD of three to five biological replicates performed in triplicates. Mann-Whitney test was used to calculate p-values between the agonists in the presence or absence of inhibitor. J-Lat-WT and J-Lat-ΔMAVS clones were treated with Catechol **(H)**, Ingenol or Bryostatin-1 **(I)** and superoxide generation was assessed by flow cytometry up to 5 hours post-treatment. Data are represented as mean ± SD of one of three biological replicate perform in triplicates. **(J)** Primary cultured T_CM_ cells generated from 6 donors were treated with Bryostatin-1, Ingenol, or TBHP for the times indicated. Cells were stained using dihydroethidium and analyzed by flow cytometry. Horizontal line indicates mean. One column t-test relative to untreated control was used to calculate p-values. **(K)** Total memory CD4+ T cells were isolated from PBMCs from 10 healthy donors. Cells were treated with Bryostatin-1, Ingenol, or TBHP for the times indicated. Cells were stained using dihydroethidium and analyzed by flow cytometry. Horizontal line indicates mean. One column t-test relative to untreated control was used to calculate p-values. Latently infected cultured T_CM_ cells from 6 donors were preincubated for 1 hour with either Gö6983 or NAC and then treated with Bryostatin-1 **(L)** or Ingenol **(M)**. %p24 positive CD4 negative cells over IL-2 control were measured 48 hours post treatment by flow cytometry. Horizontal line indicates mean. Wilcoxon matched-pairs signed rank test relative to no inhibitor (None) control was used to calculate p-values. (*p < 0.05; **p < 0.01; ***p < 0.001). ns, not significant.

To address whether MAVS is upstream or downstream of PKC activation, WT and ΔMAVS clones were pre-treated with the global PKC inhibitor Gö6983 prior to stimulation with Ingenol or Bryostatin-1 (44). Gö6983 completely abrogated viral reactivation mediated by Ingenol and Bryostatin-1 in both cell lines (**Figures 6A, B**). These results suggest that MAVS is downstream of PKC activation. There are 12 PKC isoforms belonging to three different classes based on the requirements for diacylglycerol (DAG) and/or calcium (Ca^2+^) (45). Ingenol and Bryostatin-1 can activate different PKC isoforms. Ingenol activates only PKCδ, one of the novel isoforms that only require DAG for activation (46–48). On the other hand, Bryostatin-1 can activate PKCα, a classic PKC that requires DAG and Ca^2+^ (49, 50). To address whether Ca^2+^ was required for viral reactivation, cells were pre-incubated with the Ca^2+^ chelator 1,2-bis(o-aminophenoxy)ethane-N,N,N’,N’-tetraacetic acid (BAPTA). BAPTA did not interfere with the ability of Ingenol to reactivate latent virus in both cell lines (**Figure 6C**). On the other hand, BAPTA significantly reduced the ability of Bryostatin-1 to reactivate in both cell lines (**Figure 6D**). These results indicate that Bryostatin-1 requires Ca^2+^ for optimal reactivation, while Ingenol does not. Ca^2+^ is not only a second messenger for the activation of PKC but is also required for the activation of the transcription factor Nuclear Factor of Activated T cells (NFAT). NFAT has been shown to also play a role in viral reactivation from latency (35, 51). To address whether NFAT played a role in viral reactivation induced by Ingenol or Bryostatin-1, cells were pre-incubated with the NFAT inhibitor FK506 (Tacrolimus). FK506 inhibits calcineurin, a phosphatase required for the activation of NFAT (52). This inhibitor did not interfere with the viral reactivation mediated by Ingenol or Bryostatin-1 (**Supplementary Figure 4**).

Generation of ROS and activation of the AOS has been proposed as the mechanism by which Dynasore activates MAVS (32). To confirm that ROS was necessary for viral reactivation mediated by the catechol group of Dynasore, we used the antioxidant N-acetylcysteine (NAC) to block ROS generation. Pre-incubation of the WT and ΔMAVS clones with NAC inhibited viral reactivation mediated by the catechol group in both clones (**Figure 6E**), confirming that ROS was necessary for viral reactivation. Furthermore, catechol may be able to initiate an alternative pathway of viral reactivation mediated by ROS that is independent of MAVS. We then wanted to confirm whether ROS generation was also required for the LRA activity of Ingenol and Bryostatin-1. PKC activation can lead to the activation of the NAPDH oxidase and the generation of ROS (53). Pre-incubation of cells with NAC blocked viral reactivation mediated by Ingenol in the WT clone to levels observed in the ΔMAVS clone (**Figure 6F**). For Bryostatin-1, NAC also blocked viral reactivation in the WT clone to levels observed in ΔMAVS (**Figure 6G**). NAC further reduced viral reactivation observed in the ΔMAVS clone, suggesting that Bryostatin-1, similar to Catechol, may be able to initiate an alternative pathway of viral reactivation mediated by ROS that is independent of MAVS.

Next, we wanted to address whether MAVS was required for ROS generation or whether ROS generation by Catechol, Ingenol and Bryostatin-1 precedes MAVS activation and viral reactivation. To that end, we measured ROS levels using a dihydroethidium dye. Superoxide (a type of ROS) specifically interacts with the non-fluorescent dihydroethidium and yields a fluorescent 2-hydroxyethidium product, thus allowing the detection of superoxide generation in live cells by flow cytometry (54). We observed that induction of ROS by Catechol, Ingenol and Bryostatin-1 was independent of the presence of MAVS. We observed peak ROS production at 3 hours for Catechol, and 2 hours for PKC agonists Ingenol and Bryostatin-1 (**Figures 6H, I**).

We next wished to confirm whether Ingenol and Bryostatin-1 could also induce ROS in primary CD4 T cells and thus participate in viral reactivation. First, we measured whether Ingenol and Bryostatin-1 could induce the accumulation of ROS in the cultured T_CM_ model of latency. To that end, cultured T_CM_ cells were stimulated with either Bryostatin-1, Ingenol or tert-butyl hydroperoxide (TBHP) as a positive control and ROS generation was measured at 2- and 3- hours post-stimulation. As shown in **Figure 6J**, both Bryostatin-1 and Ingenol induced the accumulation of ROS in cultured T_CM_ over untreated cells (dotted line) but to a lesser extent than the positive control TBHP. Next, we addressed whether the accumulation of ROS mediated by these two PKC agonists could be observed in memory CD4 T cells isolated from peripheral blood. ROS generation in memory CD4 T cells was measured at 2- and 3-hours post-stimulation with either Bryostatin-1, Ingenol or TBHP. The two PKC agonists induced accumulation of ROS in memory CD4 T cells comparable to the positive control TBHP (**Figure 6K**). Finally, we addressed whether ROS-induction mediated by these PKC agonists was also necessary for efficient viral reactivation in the latently infected cultured T_CM_ cells. Latently infected cells were reactivated with either Bryostatin-1 or Ingenol in the presence of the PKC inhibitor Gö6983 or the ROS scavenger NAC. As expected, Gö6983 completely abrogated viral reactivation mediated by Bryostatin-1 or Ingenol (**Figures 6L, M**). On the other hand, NAC had a partial inhibitory effect in some donors with Bryostatin-1 and significantly reduced viral reactivation mediated by Ingenol (**Figures 6L, M** respectively). In conclusion, PKC agonists induce the accumulation of ROS in primary CD4 T cells and the induction of the AOS response by PKC agonists is part of the mechanism involved in viral reactivation mediated by these two agonists.

### MAVS Is Not Necessary for HDAC Inhibitors (HDACi) or Bromodomain Inhibitor-Mediated Viral Reactivation

Next, we wanted to evaluate whether MAVS was required for the latency reversal activity of other previously described LRAs with mechanisms of action that do not involve NF-κB activation. We tested the HDACi suberoylanilide hydroxamic acid (SAHA/Vorinostat), Romidepsin and Panobinostat. These three HDACi reactivate HIV-1 by increasing histone acetylation, relaxing the chromatin structure surrounding the HIV-1 LTR and promoting the release of RNA polymerase from a paused state (55–57). SAHA or Panobinostat were able to reactivate latent HIV-1 at the same degree in the WT and ΔMAVS clones (**Supplementary Figure 5A**). However, we observed a defect in the ability of Romidepsin to reactivate latent HIV-1 in the absence of MAVS. However, this defect was not rescued when cells were complemented with MAVS (**Supplementary Figure 5B**). Finally, we tested the bromodomain inhibitors JQ-1, PFI-1, and Bromosporine. Bromodomain inhibitors reactivate latent HIV-1 by displacing BRD4 from the p-TEFb complex and allowing a stronger interaction of Tat with CyclinT1 (58–60). Although a defect in viral reactivation was observed with all these agonists, the activity of these three LRAs was not rescued by MAVS complementation (**Supplementary Figures 5C, D**). These results suggest that the defect observed with Romidepsin and bromodomain inhibitors may be due to either the clonal selection or an off-target effect of the CRISPR/Cas9 strategy and that MAVS does not play a role in the latency reversal activity of these two classes of LRAs.

### Role of MiniMAVS Isoform in ROS-Mediated Viral Reactivation

**Figure 7:**
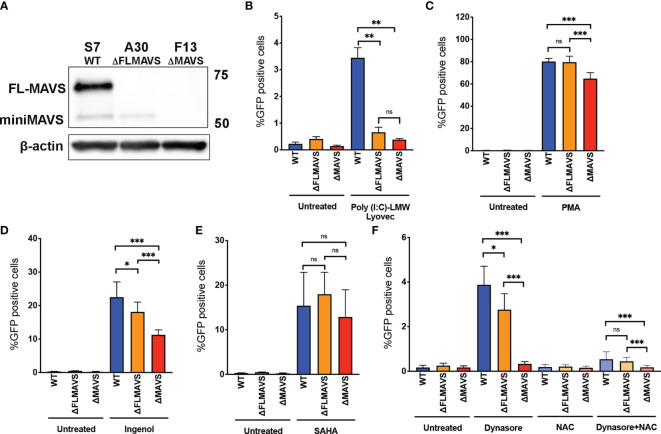
miniMAVS plays a role in the ROS-mediated reactivation of latent HIV-1. **(A)** Immunoblot of J-Lat CRISPR clones S7, A30, and F13. J-Lat CRISPR clones were incubated with RIG-I agonist Poly(I:C) LMW Lyovec **(B)**, PMA **(C)**, Ingenol **(D)**, SAHA **(E)** or Dynasore in the presence or absence of NAC **(F)**. % GFP positive cells were measured by flow cytometry. Data are represented as mean ± SD. (*p < 0.05; **p < 0.01; ***p < 0.001; ns, not significant).

As mentioned previously, MAVS is bicistronic and can give rise to two functionally distinct protein isoforms named full-length MAVS and miniMAVS (**Figure 3A**) (15, 16, 61). To further characterize the role that MAVS isoforms play in ROS-induced viral reactivation, we designed a lentiviral vector with a single gRNA targeting exon 1 of MAVS (denoted as the “A” clone, **Figure 3A**). We generated two new clones, one lacking the full-length MAVS isoform but still able to express miniMAVS (termed ΔFLMAVS), and the other one lacking both isoforms ΔMAVS as shown previously (**Figure 7A**). When we incubated the WT, ΔFLMAVS and ΔMAVS J-lat clones with Poly(I:C) LMW-Lyovec, viral reactivation was only observed in the WT clone but not the ΔFLMAVS or ΔMAVS clones (**Figure 7B**). This was expected, as the ΔFLMAVS and ΔMAVS clones lack expression of the full-length isoform that encodes the CARD domain, needed to interact with RIG-I/MDA-5. Next, we tested whether viral reactivation mediated by the PKC activator PMA required the full length or the miniMAVS isoform. Reactivation was impaired in the absence of both isoforms (ΔMAVS), but unaffected in the WT and ΔFLMAVS clone (**Figure 7C**), suggesting that only the miniMAVS isoform was necessary for efficient PMA-mediated reactivation. We next assessed the activity of Ingenol in the three clones. Lack of FLMAVS (ΔFLMAVS) resulted in a modest reduction on viral reactivation mediated by Ingenol, but absence of both MAVS isoforms (ΔMAVS) decreased viral reactivation about 50% (**Figure 7D**). These results suggests that miniMAVS is sufficient to rescue the ability of Ingenol to reactivate latent HIV. We also tested the HDACi SAHA and found its latency reversal activity to be unaffected by the presence of either MAVS isoform (**Figure 7E**). Finally, we tested Dynasore to characterize the role that miniMAVS plays in the response to ROS-mediated reactivation and the AOS response. As we previously observed, there was a reduction in viral reactivation when both MAVS isoforms were absent (ΔMAVS), but miniMAVS expression (ΔFLMAVS) was sufficient to trigger Dynasore-mediated reactivation (**Figure 7F**); and reactivation was dependent on ROS production as NAC abrogated viral reactivation (**Figure 7F**). In conclusion, we demonstrated the role of ROS and the AOS response in viral reactivation mediated by Dynasore and that miniMAVS was sufficient to initiate this AOS response but the presence of FLMAVS may be required for complete rescue. It is also worth noting that the ΔMAVS clone used in these experiments (clone F13) is a separate clone to the one used in **Figures 3–6** (clone F2). However, both F2 and F13 clones behave similarly when challenged to different LRAs, further supporting the role of MAVS in viral reactivation mediated by the ROS-inducing agent Dynasore, as well as PKC agonists.

## Discussion

**Figure 8:**
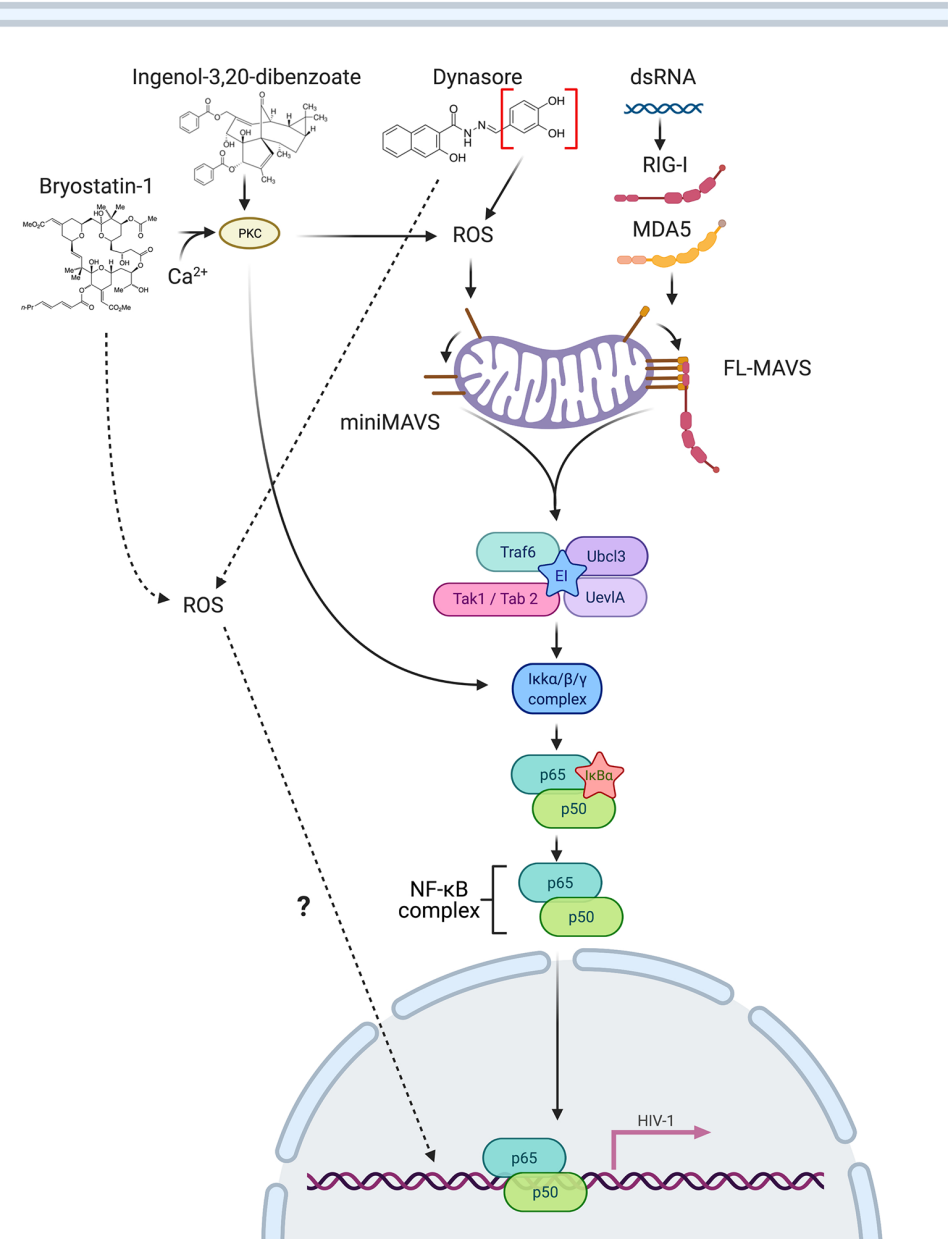
Schematic of MAVS involvement in reactivation of latent HIV-1. RIG-I/MDA5 ligands bind to RIG-I/MDA5 and directly interact with full length MAVS, inducing NF-κB activation. The PKC agonists Bryostatin-1 and Ingenol can trigger NF-κB activation by activating PKC and by inducing ROS, which activates miniMAVS, leading to NF-κB activation, translocation to the nucleus and promoting latency reversal. Dynasore or Catechol mediate ROS generation, activating miniMAVS and causing NF-κB activation. Created with BioRender.com

Recognition of cytoplasmic viral RNA by pattern recognition receptors RIG-1 and MDA5 induces a complex cascade of signaling that converges in the recruitment of MAVS/IPS-1/Cardif/VISA. This signaling results in the activation of NF-κB and IRF transcription factors in order to initiate an innate immune response to control viral infections (61–65). In this work, we demonstrated that different isoforms of MAVS also have an important role in HIV-1 reactivation from latency in CD4 T cells mediated by RIG-I ligands, PKC agonists and ROS-inducing agents. When latently infected cells are stimulated by RIG-I/MDA5 ligands, full length-MAVS interacts with ligand-bound RIG-I/MDA5 and causes activation of NF-κB causing viral reactivation (**Figure 8**). On the other hand, when latently infected cells are stimulated with the PKC agonists Bryostatin-1 or Ingenol or the ROS-inducing agents Dynasore or its active group catechol, ROS generation induces signaling through miniMAVS and promotes IkB-α degradation and subsequent activation of the NF-κB complex p65/p50 (**Figure 8**). NF-κB then binds to the viral LTR and promotes transcription of the viral genome. Finally, we also observed that Bryostatin-1 and the catechol group of Dynasore may be able to initiate an alternative pathway of viral reactivation mediated by ROS that is independent of MAVS by an unknown mechanism (**Figure 8**)

Although a plethora of reports have investigated the potential use of TLR agonists as LRAs, little effort has gone towards addressing whether the RLR RIG-I or MDA-5 can be potential targets for LRA development. To our knowledge, there are not small molecules that can specifically target RIG-I or MDA-5. Our results demonstrate that pharmacological activation of MAVS can be achieved with ROS-inducing agents such as Dynasore through the activation of the AOS response. As such, pharmacological activation of MAVS may represent a novel target toward reactivation of the latent reservoir. We have shown that Dynasore, through its catechol group, can elicit an AOS response that leads to viral reactivation from latency in CD4 T cells in a MAVS-dependent manner. This process is independent of Dynasore’s previously identified targets, dynamin and drp1. Furthermore, we demonstrate that the latency reversal activity of two well-characterized PKC agonists, Ingenol and Bryostatin-1, partially depends on the induction of ROS and the presence of MAVS. The role of MAVS and ROS in Ingenol or Bryostatin-1-mediated viral reactivation had not been described prior to this study. Our results agree with previously published studies showing that ROS-mediated signaling reactivates latent HIV-1. The treatment of the cell line Jurkat with H_2_O_2_, a ROS generator, increased activation of NF-κB and induced HIV-1 LTR-driven luciferase (66). Using a primary cell model of latency, Yang et al. discovered that 5-hydroxynaphthalene-1,4-dione (5HN, juglone) reactivated latent HIV-1 by increasing ROS and activating NF-κB (67). These studies did not investigate whether H_2_O_2_ or Juglone-mediated reactivation was MAVS-dependent, however, based on the results shown here we speculate that MAVS may play a role in both.

Our study is not without caveats. First, our primary model of latency used to evaluate viral reactivation is based on the generation of T_CM_ in culture and therefore it does not speak to how other T cell subsets may respond to ROS-inducing agents. The expression of miniMAVS and MAVS isoforms in cell reservoirs other than CD4 T_CM_ cells may be different and therefore alter the response to this type of LRAs (68–72). Furthermore, whether RIG-I agonists or Dynasore can reactivate latent HIV in macrophages is unknown. Recently, it has been shown that other PRR ligands can modulate HIV transcription in macrophages (73). This study only evaluates TLR agonists. Interestingly, activation of different TLRs either induces or represses HIV transcription. It will be interesting to evaluate whether RIG-I agonists or Dynasore will also alter HIV transcription in this cell type. Second, the model of latency used to elucidate the mechanism by which ROS induce viral reactivation is a transformed cell line modified using CRISPR/Cas9, which may not fully recapitulate all aspects of HIV latency *in vivo* and be subject to off-target effects of CRISPR technology. To strengthen our findings in the J-Lat CRISPR cell line, we confirmed the effect of ROS on viral reactivation in a primary model of latency (**Figure 6**). Additionally, our findings were confirmed using two different CRISPR ΔMAVS (clones F2 and F13) and by MAVS complementation studies. These complementation studies are critical to confirm any finding with a clonal cell line generated using CRISPR/Cas9 due to potential off-target effects of the gene editing tool or the selection process, as we observed with HDACi and bromodomain inhibitors (**Supplementary Figure 5**). Third, we observed an overall reduction in the translocation of the active subunit of NF-κB, p65, which would suggest an overall reduction in the available p65 to bind to the HIV-1 LTR and promote viral transcription. We did not show direct binding of p65 to the viral LTR. However, the role of p65 as an HIV transcription factor has been extensively studied (74). Instead, we used a combination of nuclear translocation assays and NF-κB inhibitors using our CRISPR J-Lat clones and measured outputs of viral reactivation in response to the various conditions. We show that optimal p65 translocation and subsequently, optimal viral reactivation, occurs when MAVS is present. Lastly, although viral reactivation induced by Dynasore is not as potent as αCD3/αCD28 in cells isolated from aviremic PLWH, we did observe a broader response than with other LRAs such as Panobinostat or JQ-1. Dynasore did not induce global T cell activation or toxicity in primary CD4T cells *in vitro*. Interestingly, Dynasore is more toxic in the transformed cell lines. This is probably due to their already higher ROS content than in primary CD4T cells (**Figure 3C**, CellRox). Additionally, as Dynasore has been safely administered in mice (75), it opens the possibility of further testing Dynasore either alone or in combination with other LRAs in animal models of HIV-1 latency. Thus, the use of ROS-inducing agents may be a favorable albeit mild way to reactivate latent HIV-1 that may require multiple stimulations to induce the majority of latently infected cells *in vivo*.

In conclusion, MAVS plays a central role in mediating signaling from RIG-I and MDA5 and inducing an AOS response to combat oxidative stress and this signaling pathways could be also exploited to reverse HIV-1 latency. Targeting the MAVS-NF-κB axis represents a novel and alternative therapeutic target toward reversing HIV-1 latency.

## Data Availability Statement

The raw data supporting the conclusions of this article will be made available by the authors, without undue reservation.

## Ethics Statement

These studies are covered under the institutional review board (IRB) #67637 and #58246 protocol approved by the University of Utah Institutional Review Board. The patients/participants provided their written informed consent to participate in this study.

## Author Contributions

Conceptualization: IS, CN, and AB. Methodology: IS, CN, AM, JK, HT, RF. Formal Analysis IS, CN, HT, RF, AS, AB. Investigation: IS, CN, HT, RF, RN. Resources: BS, HS, CH, AD-S. Writing – Original Draft: IS, CN, AB. Writing – Review and Editing: IS, CN, HT, LT, AS, AB. Visualization: IS, CN, HT, RF, AB. Supervision: AS, LT, VP, AB. Project Administration: AB. Funding Acquisition: CH, VP, AB. All authors contributed to the article and approved the submitted version.

## Funding

This work was partially supported by the National Institute of Health grants R21-AI106438 and R01-AI124722 to AB, R01-AI143567, R33AI122377 and R21-AI123035 to VP, cooperative agreement W81XWH-07-2-0067 between the Henry M. Jackson Foundation and the U.S. Department of Defense to LT, and P50AI150464 to CH and HS. IS was supported with a fellowship by the National Institutes of Health under the Ruth L. Kirschstein National Research Service Award NIH F31 AI147814. We thank Dr. Ryan O’Connell (University of Utah) for the lentiCRISPRv2 plasmid, Dr. Jonathan Karn (Case Western Reserve University) for the 2D10 cells, Dr. Eric Verdin (University of California San Francisco) for the J-Lat 10.6 and 6.3 cell clones, Dr. Warner Greene (Gladstone Institute) for the 5A8 cells. This work was supported by the University of Utah Flow Cytometry Facility in addition to the National Cancer Institute through Award Number 5P30CA042014-24. This research has been facilitated by the services and resources provided by the District of Columbia Center for AIDS Research, an NIH funded program (AI117970), which is supported by the following NIH Co-Funding and Participating Institutes and Centers: NIAID, NCI, NICHD, NHLBI, NIDA, NIMH, NIA, FIC, NIGMS, NIDDK, and OAR.

## Disclaimer

The content is solely the responsibility of the authors and does not necessary represent the official views of the NIH, the U.S. Army, or the Department of Defense.

## Conflict of Interest

The authors declare that the research was conducted in the absence of any commercial or financial relationships that could be construed as a potential conflict of interest.
