# Supplementary material for: Activation of the Anti-Oxidative Stress Response Reactivates Latent HIV-1 Through the Mitochondrial Antiviral Signaling Protein Isoform MiniMAVS
Source: Front Immunol. 2021 Jun 14;12:682182. doi: 10.3389/fimmu.2021.682182 (PMC8236643; doi:10.3389/fimmu.2021.682182)
Supplement: Supplementary file 1 [file DataSheet_1.docx]

Supplementary Material


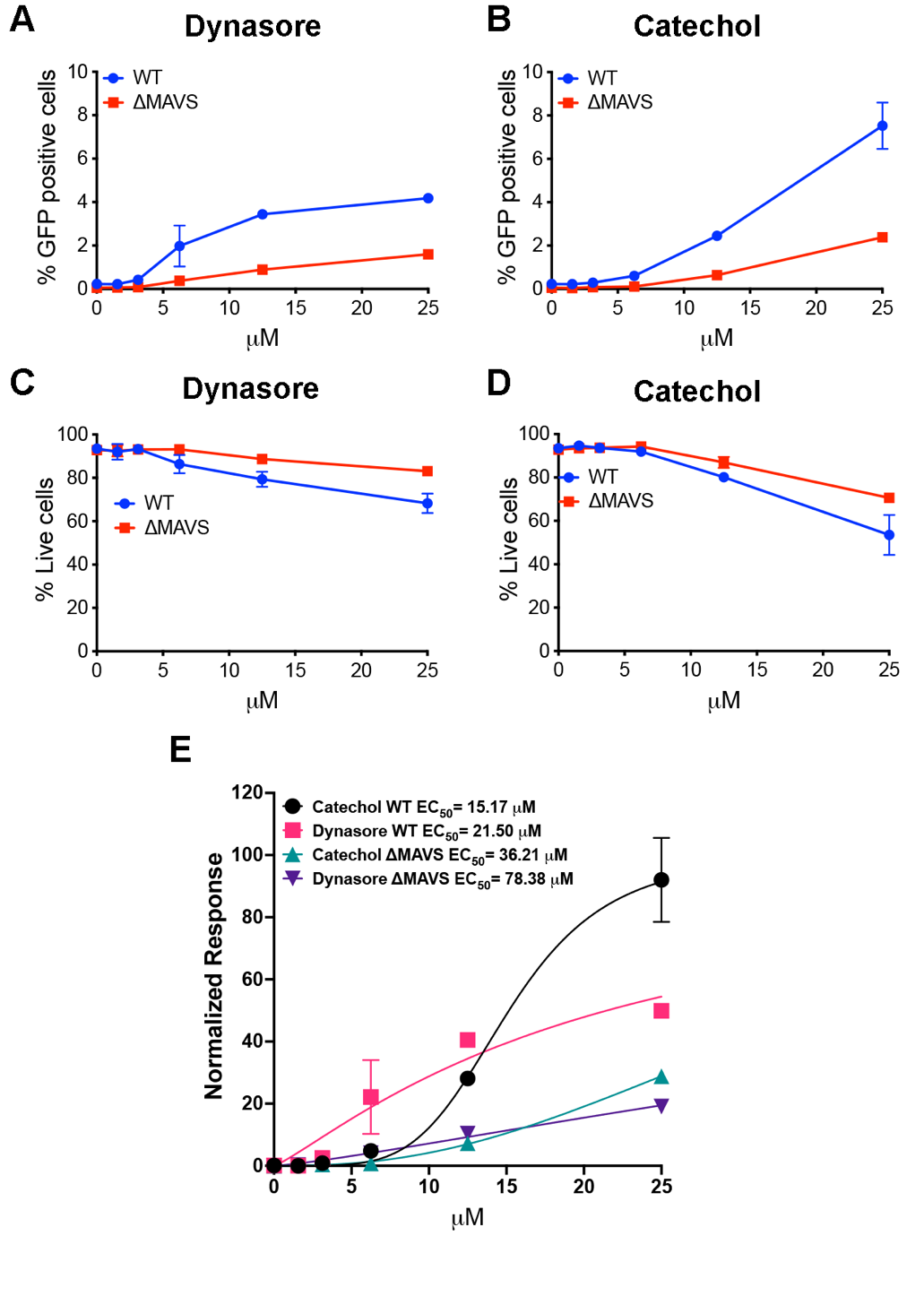


Supplementary Figure 1. Dose response of Dynasore and Catechol. J-Lat-WT and J-Lat-ΔMAVS clones were tested in their ability to reactivate latent HIV-1 with Dynasore (A and C) or Catechol (B and D). % GFP positive cells (A-B) and viability (C-D) were measured by flow cytometry. (E) EC_50_ determination for Dynasore and Catechol in J-Lat-WT and J-Lat-ΔMAVS clones. Response was normalized to maximal response with Catechol at 25 μM.


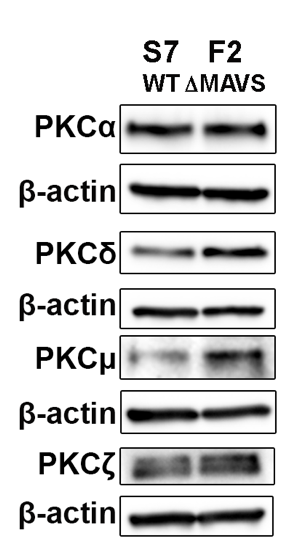


Supplementary Figure 2. PKC expression in J-Lat clones. (A) Levels of PKCα, PKCδ, PKCμ and PKCζ in J-Lat-WT and J-Lat-ΔMAVS clones.

**
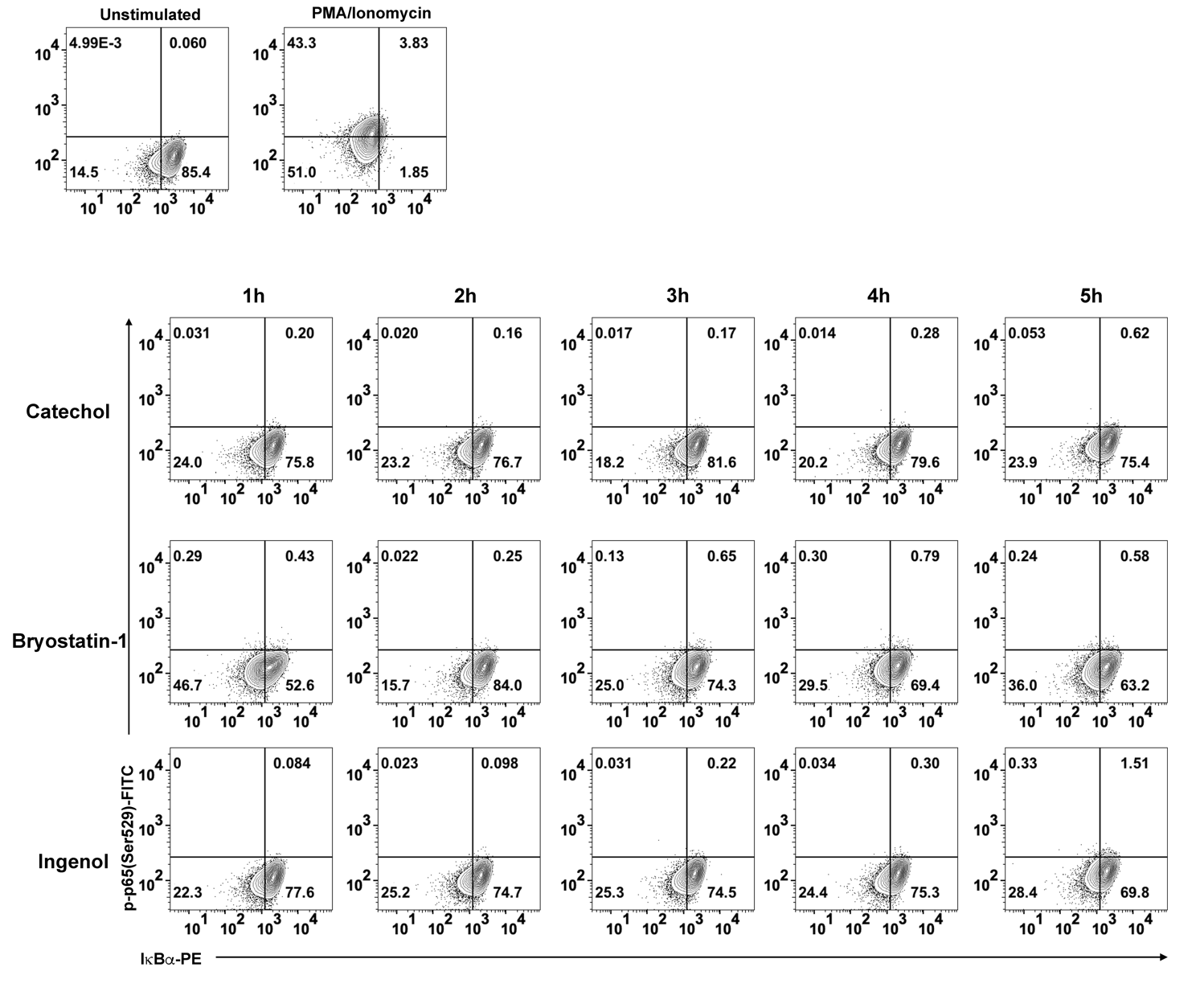
**

Supplementary Figure 3. Levels of IκB-α and p65 Ser529 phosphorylation. Representative flow plots of the levels of IκB-α and p65 Ser529 phosphorylation upon stimulation of memory CD4T cells with either Catechol, Bryostatin-1 or Ingenol at different time points. A combination of PMA and Ionomycin was used as positive control.


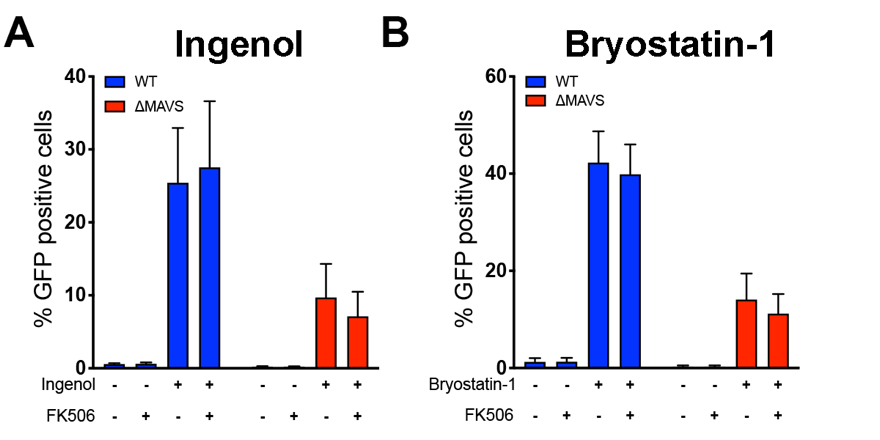


**Supplementary Figure 4.** **Viral reactivation mediated by Ingenol and Bryostatin-1 is independent of NFAT.** J-Lat-WT and J-Lat-ΔMAVS clones were tested in their ability to reactivate latent HIV-1 with Ingenol (A) or Bryostatin-1 (B) in the presence of the NFAT inhibitor FK506. % GFP positive cells were measured by flow cytometry. Data are represented as mean ± SD of three to five biological replicates performed in triplicates.


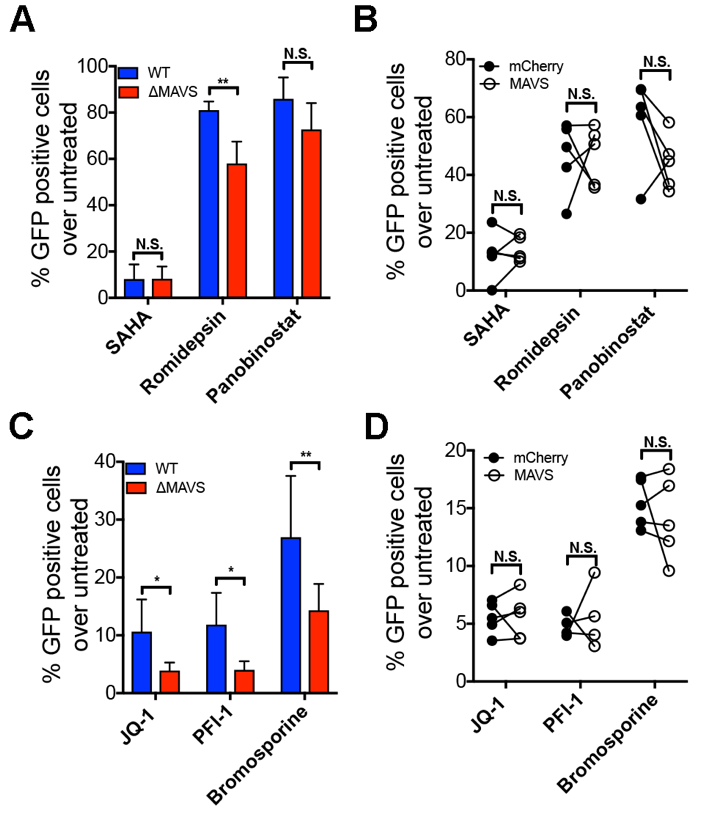


Supplemental Figure 5. MAVS does not play a role in viral reactivation mediated by HDACi or bromodomain inhibitors. J-Lat-WT and J-Lat-ΔMAVS clones were tested in their ability to reactivate latent HIV-1 with HDACi SAHA, Romidepsin, Panobinostat (A) or bromodomain inhibitors JQ-1, PFI-1, or Bromosporine (C). % GFP positive cells were measured by flow cytometry. MAVS-transduced or control mCherry-transduced J-Lat-ΔMAVS cells were treated for 24 hours with either SAHA, Romidepsin and Panobinostat (B), or JQ-1, PFI-1, or Bromosporine (D). % GFP positive cells were measured by flow cytometry. Data are represented as mean at least three biological replicates performed in triplicates. Two-sample paired t-test analysis was used to calculate p-values between MAVS-transduced or control mCherry-transduced J-Lat-ΔMAVS cells.
